# Social capital in relation to depression, musculoskeletal pain, and psychosomatic symptoms: a cross-sectional study of a large population-based cohort of Swedish adolescents

**DOI:** 10.1186/1471-2458-10-715

**Published:** 2010-11-20

**Authors:** Cecilia Åslund, Bengt Starrin, Kent W Nilsson

**Affiliations:** 1 Centre for Clinical Research, Uppsala University, Central Hospital of Västerås, S-72189 Västerås, Sweden; 2 Department of Social Studies, Karlstad University, Karlstad, Sweden; 3 Faculty of Health and Social Studies, Lillehammer College, Lillehammer, Norway

## Abstract

**Background:**

Social capital has lately received much attention in health research. The present study investigated whether two measures of subjective social capital were related to psychosomatic symptoms, musculoskeletal pain, and depression in a large population of Swedish adolescents.

**Methods:**

A total of 7757 13-18 year old students anonymously completed the Survey of Adolescent Life in Vestmanland 2008 which included questions on sociodemographic background, neighbourhood social capital, general social trust, and ill health.

**Results:**

Low neighbourhood social capital and low general social trust were associated with higher rates of psychosomatic symptoms, musculoskeletal pain, and depression. Individuals with low general social trust had more than three times increased odds of being depressed, three times increased odds of having many psychosomatic symptoms, and double the odds of having many symptoms of musculoskeletal pain.

**Conclusions:**

The findings make an important contribution to the social capital - health debate by demonstrating relations between social capital factors and self-reported ill health in a young population.

## Background

Mental ill health has been designated as a "global burden of disease", with depression accounting for most of the burden, involving large economic and social costs for society and affected individuals [1-3]. Another problem is the high prevalence of musculoskeletal and psychosomatic symptoms among young people in western societies [4,5]. Depression, musculoskeletal pain and psychosomatic symptoms that appear during adolescence often persist into adulthood and may partly be explained by psychosocial and lifestyle factors [6-8]. A shift of focus from curative to preventive measures regarding mental ill health has, through multi-disciplinary enquiry into psychosocial mechanisms, shed light upon the importance of social support, socioeconomic status and social capital [9]. Although the concept of social capital has been discussed and debated among sociologists for decades, its importance and influence has recently aroused new interest within the research field of medicine and population health.

Most research on social capital refers to the original definitions of the concept made by Bourdieu [10], Coleman [11], and Putnam [12,13], following the social school of Emile Durkheim [14]. Social participation, collective action, and co-operation for mutual benefit in informal and formal networks have been described as the core of the social capital concept [12,13,15]. This concept can, for example, be applied to neighbourhoods, workplaces, and special interest groups. Common indicators of social capital are social networks and social support, involvement in associations and politics, and measures of trust between individuals [12,16,17]. Szreter and Woolcock further developed the concept by presenting three different forms of social capital: bonding, bridging, and linking [18]. Bonding social capital refers to: "trusting and co-operative relations between members of a network who see themselves as being similar in terms of their shared social identity". Bridging social capital refers to: "relations of respect and mutuality between people who know that they are not alike in some socio-demographic (or social identity) sense (differing by age, ethnic group, class, etc)". Linking social capital refers to: "norms of respect and networks of trusting relationships between people who are interacting across explicit, formal or institutionalized power or authority gradients in society".

Social capital has, among other things, been related to mental ill health [19-21], general ill health [17,22,23], mortality [24], and violent crime [25]. The social gradient of health, i.e. the strong association between low socioeconomic status and an elevated risk for disease and premature death in western economies [26], as well as the associations between large income inequalities within a society and high rates of ill health [27] have been suggested to be dependent on social capital as a mediating factor, presumably by socioeconomic inequality resulting in less trusting and reciprocal relationships between individuals and lower levels of social cohesion and civic and political participation [25,28-30]. Kawachi et al [15] found evidence suggesting that the relationship between inequality and mortality among the different states of the USA is mediated by social trust, a dimension of social capital which refers to the proportion of citizens who agree with statements such as: "most people can be trusted" or "most people would try to take advantage of you if they got a chance". Social environments marked by wide income disparities generate invidious social comparisons which create a sense of exclusion and alienation among vulnerable individuals [25]. Such environments create experiences of shame, inferiority, subordination, and disrespect which are common sources of anxiety and psychological stress [31], emotions that through neuroendocrine pathways may be translated into physical manifestations of ill health [28,32-36].

Most previous studies of social capital and ill health have focused on health in adult populations, and less is known about how social capital may influence health among adolescents. This age group may be particularly affected by social capital as a cause of limited mobility [37] and have an increased vulnerability to feelings of shame and status due to greater sensitivity to peer evaluation and physical and cognitive maturational changes [38]. In 1998, Stevenson found a relation between neighbourhood social capital and depression among African American adolescents [39]. These findings were confirmed in a later study of African American youth living in a high-poverty inner city area [37]. In a cross-national study, Drukker et al found that higher levels of social cohesion and trust were associated with higher levels of perceived health among adolescents [40]. In another study the same research group found associations between social capital aggregated to the neighbourhood level and health, but only in children 11 years or younger [41,42]. Associations between high social capital and low levels of psychological distress have also been found in a study of Australian adolescents [20], although another Australian study did not find any independent effect of area-level social capital and self-rated adolescent health [29]. Two recent studies found associations between low levels of neighbourhood social capital and worse perception of health [43] as well as social capital in school and self-reported health [44] among adolescents. Few of these studies, however, used diagnostic instruments in order to evaluate the health of the participants. Instead, they used subjective perception of overall general health as measurement [29,40,43,44]. There is a need to examine the associations between social capital and more specific health problems among adolescents.

We aimed to determine whether two forms of bonding and bridging social capital, in this study referred to as "neighbourhood social capital" (such as neighbourhood connections and trust, reciprocity and feelings of safety) and "general social trust" (such as how well the participants perceive that people in general can be trusted), were associated with self-reported health in a large population of Swedish adolescents. We hypothesised that low neighbourhood social capital and low general social trust would be associated with higher rates of psychosomatic symptoms, musculoskeletal pain, and depression.

## Methods

The present study was part of the Survey of Adolescent Life in Vestmanland 2008 (Salve-2008), a survey distributed biannually by the County Council of Västmanland, Sweden. Västmanland is a medium-sized county situated approximately 100 km west of Stockholm. All school students in 7^th ^(13-14 years old) and 9^th ^(15-16 years old) grade of elementary school, and 2^nd ^(17-18 years old) grade of secondary school in the county, a total of 57 schools, were asked to complete a questionnaire during class hours. The questionnaire included questions about demographic background, neighbourhood social capital, general social trust, and psychosomatic, musculoskeletal and depressive symptoms.

A total of 7906 students completed the questionnaire, comprising 78.2% of the total population. The exclusion of 41 participants who did not state their sex, and 108 participants who did not complete the questionnaire satisfactorily, left 7757 participants for the analyses. There were 1351 boys and 1394 girls in 7^th ^grade, 1291 boys and 1314 girls in 9^th ^grade, and 1230 boys and 1177 girls in 2^nd ^grade of secondary school. Participation was anonymous and voluntary. The study followed the Swedish guidelines for studies of social science and humanities according to the Declaration of Helsinki. According to Swedish regulations, this type of study no longer applies for ethical approval by the medical faculty.

### Measures

*Sex*, whether the participant was a boy (0) or a girl (1).

*Parental unemployment*, whether both parents were working (0) or one or both parents were unemployed (1). In Sweden, the most common family constellation involves both parents working. Families where one of the parents is a house wife or husband, and thus permanently removed from the labour market, are rather rare [45].

*Living conditions*, whether the participant was living in a single-family house (0) or a multi-family house (1).

*Ethnicity*, whether both parents were born in Sweden or Scandinavia (0) or at least one parent was born outside of Scandinavia (1).

*Subjective socioeconomic status*, where participants were asked to rank the socioeconomic status of their family on a 7-point Likert scale by the following question: "Imagine society as being like a ladder. At the bottom are those with the least money, and at the top are those with the most. If you think about how wealthy your own family is compared with the rest of society, where would you place your family on this scale?"

*Housing area*, where the participants stated their living area out of a choice of 31 defined areas of the county. Medium income statistical data for the general population of each of the 31 areas were obtained from Statistics Sweden. The variable was used as an objective measure of the socioeconomic status of the housing areas.

#### Neighbourhood social capital

Participants were asked to respond to seven statements about their neighbourhood (a part of a city, town or village): 1, In my neighbourhood, no-one needs to feel afraid. 2, It is unsafe to be outside at night in my neighbourhood. 3, In my neighbourhood, people seldom help each other. 4, If anything at home should break or go missing, we can always get help from a neighbour. 5, Most people know each other in my neighbourhood. 6, There are seldom any fights or trouble in my neighbourhood. 7, I often see graffiti and damaged objects (park benches, bus stops, street lights) in my neighbourhood. Response alternatives were: Strongly agree (1), Agree to some extent (2), Disagree to some extent (3), Strongly disagree (4). The internal consistency (Chronbach's alpha α) of the neighbourhood social capital questions was 0.71. A summation index was created with a range of 7-28 points where items 2, 3, and 7 were reversed. The index was also divided by quartiles, where the 1^st ^quartile counted as low neighbourhood social capital, the 2^nd^-3^rd ^quartiles counted as medium neighbourhood social capital, and the 4^th ^quartile counted as high neighbourhood social capital.

#### General social trust

The participants were asked to respond to six statements about how they felt about people in general: 1, Most people can be trusted. 2, You can never be too careful when meeting new people. 3, Most people try to be helpful. 4, Most people would try to use others if they had the opportunity. 5, Most people only care about themselves. 6, Most people are honest. Response alternatives were: Strongly agree (1), Agree to some extent (2), Disagree to some extent (3), Strongly disagree (4). The internal consistency of the general social trust questions was α = 0.73. A summation index was created with a range of 6-24 points, where items 2, 4 and 5 were reversed. The index was also divided by quartiles, where the 1^st ^quartile counted as low general social trust, the 2^nd^-3^rd ^quartiles counted as medium general social trust, and the 4^th ^quartile counted as high general social trust.

#### Psychosomatic symptoms

The participants were asked how often they suffered from: 1, Headache. 2, Stomach ache. 3, Feelings of nervousness. 4, Feelings of irritation. 5, Sleep problems. Response alternatives were: Never (0), Rarely (1), Sometimes (2), Often (3), Always (4). The internal consistency of the questions of psychosomatic symptoms was α = 0.75. A summation index was created with a range of 0-20 points. The index was divided by standard deviations, where -1 SD was the cut-off point for few psychosomatic symptoms, +1 SD was the cut-off for many psychosomatic symptoms, and the intermediate group was counted as having a medium number of psychosomatic symptoms. We created a dichotomous variable, with few-medium symptoms (0) and many symptoms (1).

#### Musculoskeletal pain

Participants were asked how often they suffered from: 1, Pain in the shoulders/neck. 2, Pain in the back/hips. 3, Pain in the hands/knees/legs/feet. Response alternatives were: Never (0), Rarely (1), Sometimes (2), Often (3), Always (4). The internal consistency of the questions of musculoskeletal pain was α = 0.68. A summation index was created with a range of 0-12 points. The index was divided by standard deviations, where -1 SD was the cut-off point for few musculoskeletal pain symptoms, +1 SD was the cut-off for many musculoskeletal pain symptoms and the intermediate group was counted as having a medium number of musculoskeletal pain symptoms. We created a dichotomous variable, with few-medium symptoms (0) and many symptoms (1).

#### Depressive symptomatology

Depressive symptoms were measured by the Depression Self-Rating Scale (DSRS) of the Diagnostic and Statistical Manual of Mental Disorders, 4^th ^edition (DSM-IV), A-criterion, for major depression with a reported sensitivity of 96.1% and a specificity of 59.4% for major depression [46,47]. In the DSM-IV, the A-criterion for major depression in adolescents is defined as two weeks of either dysphoric or irritable mood or loss of interest or pleasure in most activities. At least one of these general criteria must be accompanied by at least four other symptoms, including sleep disturbances, weight loss or gain/appetite disturbances, psychomotor agitation or retardation, fatigue or loss of energy, feelings of worthlessness or guilt, concentration disturbances, and thoughts of suicide. The internal consistency of the questions of depression was α = 0.83. For the analyses, a depression index was calculated as a summation of the reported symptoms according to the DSRS scale, each symptom counting only once (0-9 points). A dichotomous variable was also created where subjects fulfilling the DSM-IV A-criterion were classified as depressed.

### Statistical analyses

Sex differences in the demographic data as well as differences in outcomes related to demographic background were analysed by Pearson's χ^2^. Sex differences in socioeconomic status and the dependent and independent factors of the study were analysed using the Mann-Whitney test. For analysis of correlations between psychosomatic symptoms, musculoskeletal pain, depressive symptoms, neighbourhood social capital, general social trust, subjective socioeconomic status, and housing area we used Spearman's rho. Linear and logistic regressions were used for analyses of relations between neighbourhood social capital, general social trust and the different measures of health. As neighbourhood social capital and general social trust on an individual level could be affected by intra-class correlation cluster effects, such as school and housing area, hierarchical linear and logistic regression analyses taking clustering effects into account were performed. We used random-effects regression models (RRM), in view of the fact that the number of subjects within each cluster was unbalanced [48]. In the final models, the hierarchical regression analyses were controlled for subjective socioeconomic status, type of residence, parental unemployment, and ethnicity. All analyses were made in SPSS v17.

## Results

Girls reported more psychosomatic symptoms, musculoskeletal pain and depressive symptoms, and boys reported higher neighbourhood social capital and higher general social trust (Table 1). A total of 6.4% of the boys and 20.4% of the girls were classified as having many psychosomatic symptoms (χ^2 ^= 616.23, *p *< 0.001), 9.2% of the boys and 17.3% of the girls were classified as having many musculoskeletal pain symptoms (χ^2 ^= 186.33, *p *< 0.001), and 15.9% of the boys and 32.4% of the girls fulfilled the criteria for depression (χ^2 ^= 287.17, *p *< 0.001). Regarding demographic data, 24.9% of the boys and 25.8% of the girls lived in a multifamily house (*p *= 0.389), 20.3% of the boys and 21.9% of the girls had at least one unemployed parent (*p *= 0.091), and 17.4% of the boys and 17.0% of the girls had at least one parent born outside of Scandinavia or were themselves born outside of Scandinavia (*p *= 0.661). Mean subjective socioeconomic status was higher among boys (*M *= 4.43, *95% CI *= 4.40-4.47, *SD *= 1.06) than among girls (*M *= 4.30, *95% CI *= 4.27-4.33, *SD *= 0.96, *p *< 0.001).

**Table 1:** Means, Medians (Med.), *SD*s, *95% CI*s and quartiles for the index factors of the study, split on boys and girls, with test statistics and p-values for sex differences.

	Boys					Girls						
	
	Mean	**Med**.	*SD*	*95% CI*	*Q1-* *Q3*	Mean	**Med**.	*SD*	*95% CI*	*Q1-* *Q3*	*Z*	*P*
Psychosomatic symptoms	6.86	7.00	3.51	6.75-6.97	4-9	9.36	9.00	3.48	9.25-9.47	7-12	-30.31	< 0.001

Musculoskeletal pain	3.12	3.00	2.49	3.04-3.20	1-5	4.02	4.00	2.68	3.94-4.11	2-6	-15.25	< 0.001

Depressive symptoms	2.12	1.00	2.38	2.05-2.20	0-3	3.36	3.00	2.73	3.28-3.45	1-5	-20.97	< 0.001

Neighbourhood social capital	22.08	22.00	3.79	21.96-22.20	20-25	21.80	22.00	3.75	21.68-21.92	19-25	-3.71	< 0.001

General social trust	15.22	15.00	3.01	15.12-15.32	13-17	14.87	15.00	2.90	14.77-14.96	13-17	-5.71	< 0.001

There was a medium-sized correlation between psychosomatic symptoms and musculoskeletal pain, psychosomatic symptoms and depression, and musculoskeletal pain and depression (Table 2). The neighbourhood social capital and general social trust factors showed a weak correlation to each other as well as to the indexes of psychosomatic symptoms, musculoskeletal pain, depressive symptoms, and subjective socioeconomic status (Table 2).

**Table 2:** Correlations with Spearman's rho between the factors of the study.

Factors^a^	**1**.	**2**.	**3**.	**4**.	**5**.	**6**.
**1. Psychosomatic symptoms**	1	.47	.58	-.24	-.27	-.17

**2. Musculoskeletal pain**	-	1	.37	-.17	-.19	-.10

**3. Depressive symptoms**	-	-	1	-.24	-.26	-.16

**4. Neighbourhood social capital**	-	-	-	1	.30	.18

**5. General social trust**	-	-	-	-	1	.13

**6. Subjective socioeconomic status**	-	-	-	-	-	1


^a ^All correlations *p *< = 0.001

The relations between objective socioeconomic status and neighbourhood social capital, general social trust, and subjective socioeconomic status were analysed by using household medium income data of different housing areas according to Statistics Sweden. The number of participants living in each housing area ranged from 64 to 666. Individual medium income in the county was € 18.287 (SD = € 1992), and medium income of the different housing areas ranged between € 13.815 - 24.651. The housing areas differed widely in levels of neighbourhood social capital and general social trust. Higher objective socioeconomic status of a housing area was correlated with a higher level of neighbourhood social capital (*r *= 0.204, *p *< 0.001), a higher level of general social trust (*r *= 0.055, *p *< 0.001), and a higher level of subjective socioeconomic status (*r *= 0.059, *p *< 0.001). Higher subjective socioeconomic status was, moreover, correlated with higher neighbourhood social capital and higher social trust (Table 2).

Neighbourhood social capital and general social trust on an individual level may be affected by intra-class correlation cluster effects, such as school and housing area. Therefore, hierarchical linear regression analysis taking clustering effects into account were performed. There were very similar estimates in the ordinary regression models and the multilevel models for the indexes of psychosomatic symptoms, musculoskeletal pain and depression symptoms (not shown in tables). In the final model including 2- and 3-level effects of psychosomatic symptoms, neighbourhood social capital (*β *= -0.19, *p *< 0.001) and general social trust (*β *= -0.27, *p *< 0.001), as well as the 2-level effect of individuals within schools (*β *= 0.17, *p *= 0.021) were significant. However, the 3-level effect of individuals within schools within housing area was not significant.

The final model including 2- and 3-level effects of musculoskeletal pain showed an association of neighbourhood social capital (*β *= -0.10, *p *< 0.001) and general social trust (*β *= -0.14, *p *< 0.001), as well as the 2-level effect of individuals within schools (*β *= 0.07, *p *= 0.024). The 3-level effect was not significant.

The final model including 2- and 3-level effects of depressive symptoms showed an association of neighbourhood social capital (*β *= -0.10, *p *< 0.001) and general social trust (*β *= -0.20, *p *< 0.001), as well as the 2-level effect of individuals within schools (*β *= 0.10, *p *= 0.025). The 3-level effect was not significant.

A comparison of logistic regression models in Table 3 shows that there were no major clustering effects of school or housing area. Neighbourhood social capital and general social trust showed the same odds ratios in relation to psychosomatic symptoms and musculoskeletal pain at individual level (column 1), at 2-level models (column 2 & 3) and at 3-level models (individuals nested in schools, which are nested in housing areas, column 4). There were, however, small, although noticeable, hierarchical effects of neighbourhood social capital and general social trust in relation to depression in the individual-within-housing area level, and in the 3-level models. Thus, small but detectable cluster effects depending on housing area affected neighbourhood social capital and general social trust in relation to depression. Individuals within the group with low neighbourhood social capital had approximately double the odds of having many psychosomatic symptoms, many symptoms of musculoskeletal pain, and depression compared with those with high neighbourhood social capital. Individuals within the group with low general social trust had a more than three times increased odds of depression, more than a three times increased odds of having many psychosomatic symptoms, and more than double the odds of many symptoms of musculoskeletal pain compared with the group with high general social trust.

**Table 3:** Multilevel analysis^1^.

	Many psychosomatic symptoms		Many symptoms of musculoskeletal pain		Depression	
**Individual level (Level 1), ordinary regression model**						

	*Log likelihood *= 6366.60		*Log likelihood *= 5422.22		*Log likelihood *= 7430.14	

***Neighbourhood social capital***	***OR (95% CI)***	***p***	***OR (95% CI)***	***p***	***OR (95% CI)***	***p***

High	Ref		Ref		Ref	

Medium	1.39 (1.14-1.68)	0.001	1.31 (1.06-1.62)	0.013	1.52 (1.28-1.81)	< 0.001

Low	2.49 (2.03-3.06)	< 0.001	1.92 (1.53-2.41)	< 0.001	2.79 (2.31-3.35)	< 0.001

***General social trust***						

High	Ref		Ref		Ref	

Medium	1.48 (1.20-1.83)	< 0.001	1.40 (1.11-1.76)	0.004	1.48 (1.24-1.78)	< 0.001

Low	3.39 (2.73-4.21)	< 0.001	2.64 (2.08-3.35)	< 0.001	3.38 (2.80-4.10)	< 0.001

**Individual level within school, 2-level analysis**						

	*QICC^2 ^*= 6376.60		*QICC *= 5432.22		*QICC *= 7440.14	

***Neighbourhood social capital***	***OR (95% CI)***	***p***	***OR (95% CI)***	***p***	***OR (95% CI)***	***p***

High	Ref		Ref		Ref	

Medium	1.39 (1.13-1.70)	0.002	1.31 (1.04-1.64)	0.019	1.52 (1.26-1.84)	< 0.001

Low	2.49 (2.03-3.06)	< 0.001	1.92 (1.53-2.40)	< 0.001	2.79 (2.23-3.47)	< 0.001

***General social trust***						

High	Ref		Ref		Ref	

Medium	1.48 (1.26-1.74)	< 0.001	1.40 (1.11-1.77)	0.005	1.48 (1.26-1.74)	< 0.001

Low	3.39 (2.83-4.05)	< 0.001	2.64 (2.01-3.46)	< 0.001	3.38 (2.87-3.98)	< 0.001

**Individual level within housing area, 2-level analysis**						

	*QICC *= 5939.22		*QICC *= 5018.40		*QICC *= 6949.51	

***Neighbourhood social capital***	***OR (95% CI)***	***p***	***OR (95% CI)***	***p***	***OR (95% CI)***	***p***

High	Ref		Ref		Ref	

Medium	1.42 (1.19-1.69)	< 0.001	1.32 (1.01-1.74)	0.046	1.49 (1.31-1.71)	< 0.001

Low	2.52 (2.05-3.11)	< 0.001	1.96 (1.51-2.54)	< 0.001	2.73 (2.30-3.24)	< 0.001

***General social trust***						

High	Ref		Ref		Ref	

Medium	1.52 (1.25-1.84)	< 0.001	1.39 (1.12-1.73)	0.003	1.56 (1.24-1.95)	< 0.001

Low	3.40 (2.77-4.18)	< 0.001	2.63 (2.04-3.39)	< 0.001	3.49 (2.82-4.31)	< 0.001

**3-level analysis**						

	*QICC *= 5939.22		*QICC *= 5018.40		*QICC *= 6949.51	

***Neighbourhood social capital***	***OR (95% CI)***	***p***	***OR (95% CI)***	***p***	***OR (95% CI)***	***p***

High	Ref		Ref		Ref	

Medium	1.42 (1.15-1.75)	0.001	1.32 (1.04-1.68)	0.021	1.49 (1.23-1.81)	< 0.001

Low	2.52 (2.02-3.15)	< 0.001	1.96 (1.54-2.49)	< 0.001	2.73 (2.20-3.40)	< 0.001

***General social trust***						

High	Ref		Ref		Ref	

Medium	1.52 (1.26-1.82)	< 0.001	1.39 (1.11-1.75)	0.005	1.56 (1.31-1.85)	< 0.001

Low	3.40 (2.82-4.11)	< 0.001	2.63 (2.00-3.46)	< 0.001	3.49 (2.89-4.20)	< 0.001

^1 ^The individual level is the ordinary logistic regression. Individuals in school and in housing area are hierarchical analyses controlling for non-independent associations of school and housing area, respectively. The 3-level hierarchical analysis considers individuals nested in schools which are nested in housing area.

^2 ^Corrected Quasi Likelihood under Independence Model Criterion (QICC)

The final 3-level hierarchical regression models were also adjusted for non-independent covariates, sex, parental unemployment, living conditions, ethnicity, and subjective SES, which were all significantly related to neighbourhood social capital, general social trust, psychosomatic symptoms, musculoskeletal pain and depression in univariate analyses (not shown in tables). As shown in Table 4, girls had more psychosomatic symptoms, musculoskeletal pain and depression. Parental unemployment was related to psychosomatic symptoms and musculoskeletal pain and low subjective SES was related to psychosomatic symptoms, musculoskeletal pain and depression. Furthermore, the adjustment for the non-independent variables did not alter the associations of neighbourhood social capital or general social trust in relation to psychosomatic symptoms, musculoskeletal pain and depression that were found in the non-adjusted models.

**Table 4:** Hierarchical logistic regressions on the three variable level of individuals clustered within schools and within housing areas, presenting odds ratios (*OR*), *95% CI*s, and significance levels of participants with many psychosomatic and musculoskeletal symptoms and participants who were classified as depressed, in relation to neighbourhood social capital, general social trust, sex, parental unemployment, living conditions, ethnicity, and subjective SES.

	Psychosomatic symptoms		Musculoskeletal pain		Depression	
	
	*OR (95% CI)*	*p*	*OR (95% CI)*	*p*	*OR (95% CI)*	*p*
**Neighbourhood social capital**						

High	Ref		Ref		Ref	

Medium	1.41 (1.14-1.75)	0.002	1.30 (1.03-1.66)	0.030	1.46 (1.21-1.75)	< 0.001

Low	2.29 (1.81-2.90)	< 0.001	1.74 (1.35-2.26)	< 0.001	2.36 (1.88-2.96)	< 0.001

**General social trust**						

High	Ref		Ref		Ref	

Medium	1.51 (1.24-1.85)	< 0.001	1.41 (1.12-1.77)	0.004	1.54 (1.29-1.85)	< 0.001

Low	3.20 (2.59-3.97)	< 0.001	2.51 (1.89-3.33)	< 0.001	3.34 (2.75-4.06)	< 0.001

**Sex**						

Boy	Ref		Ref		Ref	

Girl	3.77 (3.21-4.44)	< 0.001	1.90 (1.58-2.29)	< 0.001	2.59 (2.24-3.00)	< 0.001

**Parental unemployment**						

Both parents working	Ref		Ref		Ref	

At least one parent unemployed	1.28 (1.07-1.53)	0.007	1.24 (1.02-1.50)	0.031	1.26 (1.10-1.45)	0.001

**Living conditions**						

Single-family house	Ref		Ref		Ref	

Multi-family house	0.98 (0.81-1.17)	0.787	0.99 (0.81-1.20)	0.897	0.97 (0.83-1.14)	0.732

**Ethnicity**						

Scandinavian ethnicity	Ref		Ref		Ref	

Non-Scandinavian ethnicity	0.86 (0.72-1.03)	0.104	0.88 (0.72-1.08)	0.230	0.93 (0.79-1.09)	0.344

**Subjective SES**						

High	Ref		Ref		Ref	

Medium	1.00 (0.75-1.33)	0.983	1.03 (0.78-1.35)	0.851	0.88 (0.70-1.10)	0.273

Low	2.00 (1.50-2.68)	< 0.001	1.70 (1.21-2.39)	0.002	1.96 (1.52-2.51)	< 0.001

## Discussion

The present study investigated whether subjective neighbourhood social capital and general social trust were associated with self-reported health in a large population of Swedish adolescents. The main finding was that low neighbourhood social capital and low general social trust were associated with higher rates of psychosomatic symptoms, musculoskeletal pain and depression. The association between general social trust and ill health was particularly evident, where the group with low general social trust had a more than three times increased odds of depression, a more than three times increased odds of having many psychosomatic symptoms, and more than double the odds of having many symptoms of musculoskeletal pain compared with the group with high general social trust. The results were controlled for potential confounding factors such as subjective family socioeconomic status, type of residence, parental unemployment and ethnicity, as well as clustering effects of school and housing area.

It is interesting that even in a highly egalitarian country such as Sweden there is an association between neighbourhood social capital, general social trust and ill health in an adolescent population. Our results agree with previous findings of relations between social capital and ill health among adolescents [37,39,40,43]. Social capital has been suggested as one important mediating factor of the relations between income inequality and ill health, as low socioeconomic status and income inequality result in less trusting and reciprocal relationships between citizens, and lower levels of civic and political participation [25,28-30]. However, the debate regarding the concepts of social capital and social trust and how they are supposed to be measured is not settled. The different schools of sociology, either employing a consensus or a conflict perspective, or a macro or micro perspective, always attract severe criticism from the opposing side [49]. The perspectives employed by sociologists can be organized into groups based on similarities and differences in their theoretical assumptions. For example; Putnam and Bourdieu have different definitions of social capital. Putnam describes social capital by using a functionalist, consensual perspective, whereas Bourdieu's definition of social capital is based more on conflict and exploitation. We aimed to determine whether two forms of bonding and bridging social capital - in this study referred to as "neighbourhood social capital" and "general social trust" - were related to poor health among adolescents. Our measures are in line with Putnam's description of social capital, although this way of using the concept of it can always be criticised.

Several limitations of the study should be noted. Firstly, the analyses are based mainly on self-reports which involves a risk of information bias due to false or inaccurate responses from the participants. Regarding self-reports of health, girls may be more aware of, and/or more inclined to report ill health which might have influenced the higher rates of it in this group. However, since we were interested in associations between individual perspectives of neighbourhood social capital and general social trust, self-reports were deemed the most convenient choice of measurement. Secondly, there is the problem of causality regarding neighbourhood social capital, general social trust and ill health, as the cross-sectional design of the study involved no possibility to distinguish the directions of cause and effect. Individuals suffering from pain and depression may be more inclined to interpret their environment and relations to other individuals in society in a negative way, which might have influenced the associations between neighbourhood social capital, general social trust and ill health. However, when analysing levels of self-reported neighbourhood social capital and general social trust in different housing areas, these plainly differed depending on the income distribution of each housing area as obtained from Statistics Sweden. A higher objective socioeconomic status of a housing area was correlated with a higher level of neighbourhood social capital, a higher level of general social trust, and a higher level of subjective socioeconomic status. Thus, the differences in self-reported neighbourhood social capital and general social trust were associated with the objective measure of socioeconomic status and would not merely be a question of negative interpretation of social relations and society as a cause of illness.

Moreover, even though there were substantial overlaps between the dependent variables (*R^2 ^*≈ 14-34%) there were just minor correlations between the dependent and independent variables (*R^2 ^*≈ 2-6%) when analysed as simple correlations (Table 2). However, in our multivariate models we found considerable odds elevation of psychosomatic symptoms, musculoskeletal pain, and depression (30-330%) in relation to neighbourhood social capital and general social trust. We therefore interpret our findings as being in line with those previously reported among adolescents [20,37,39,40,43,44], and suggest that neighbourhood social capital and general social trust may be related to specific health diagnoses among adolescents.

Thirdly, we used different cut-off points for the outcome variables compared with the independent variables. The reason for this is that we did not want the measures of low and high neighbourhood social capital and general social trust to be too strict, and consequently used quartiles (highest and lowest 25%) as cut-offs. However, for the outcome measures of psychosomatic symptoms and musculoskeletal pain, we wanted to identify the participants with the most problems and therefore used the stricter cut-offs of standard deviations (approximately 16% of the individuals with the highest scores). However, the measurement of depression had high sensitivity and low specificity, providing a risk for false-positive classifications. The Depression Self-Rating Scale of the DSM-IV, A-criterion, involves the risk of including participants who would be ruled out according to the more strict B-, C-, D-, and E-criteria. This scale has, nevertheless, been proved to be a useful instrument for defining major depression [46]. The outcome scales were, moreover, used in two different ways: as a summation index of reported symptoms in the linear regression analyses, and as dichotomous variables for the logistic regressions. The dependent variables of the indexes of psychosomatic and musculoskeletal symptoms, as well as depression, were skewed. One way of dealing with this kind of problem would be to transform the data, e. g., by a log or log-log transformation. In the present study, however, neither the log- nor the log-log transformation produced a symmetric distribution of the data, and were therefore not optimal for parametric modelling methods. The procedure with complementary statistical approaches can help to overcome shortcomings with the individual statistical methods and help to eliminate scaling artefacts. Both the hierarchical multi-level linear regressions and the logistic regressions showed similar results, in the same direction, which added further support for the presented findings.

The present study also has several strengths, particularly regarding the large population based sample of adolescents and the high participation rate which might offer an opportunity to generalise the results to other adolescent populations as well. Most previous studies have examined relations between social capital and health in adult populations. The present study focuses on adolescents, to examine whether social capital may be associated with health outcomes among young people as well. Moreover, the few previous studies of social capital that have focused on adolescent populations have often chosen specific areas of low socioeconomic status and high deprivation for their analyses. The present study involves a large adolescent population from a county that is considered to be representative of Sweden as a whole because of its distribution of educational, income, and employment levels as well as urban and rural areas [50]. Thus, our study contributes important information to the research field by its generalizability to other adolescent populations. Further, previous studies have often used general self-rated health as an outcome, which is not a diagnostic instrument but rather a subjective feeling of whether one's health is good or bad [29,40,43,44]. The use of a validated diagnostic instrument for major depression, and specific questions regarding musculoskeletal pain and psychosomatic symptoms, allows for an evaluation of actual differences in the prevalence of specific common health problems as a complement to previous studies of subjective views of general health.

## Conclusions

Our findings suggest a link between subjective neighbourhood social capital, general social trust, and ill-health in a Swedish adolescent population. If our findings are valid, they make an important contribution to the social capital - health debate by demonstrating relations between social capital factors and self-reported ill health in an adolescent population.

## List of abbreviations

DSRS DSM-IV: Depression Self-Rating Scale of the Diagnostic and Statistical Manual of Mental Disorders, text revision, 4^th ^edition;

## Competing interests

The authors declare that they have no competing interests.

## Authors' contributions

Study concept and design: CÅ, KWN, BS. Acquisition of data: CÅ, KWN. Questionnaire management: CÅ. Analysis and interpretation of data: CÅ, KWN, BS. Drafting of manuscript: CÅ, KWN. Critical revision: CÅ, KWN, BS. All authors read and approved the final manuscript.

## Pre-publication history

The pre-publication history for this paper can be accessed here:

http://www.biomedcentral.com/1471-2458/10/715/prepub
